# Biomarkers and Risk Prediction in Cardiovascular–Kidney–Metabolic Syndrome: A Comprehensive Review

**DOI:** 10.31083/RCM50631

**Published:** 2026-09-16

**Authors:** Yuanyuan Lu, Mingxin Yang, Jinbo Yu, Pan Lin, Jun Ji, Xiaoqiang Ding, Yaqiong Wang, Xialian Xu

**Affiliations:** ^1^Department of Nephrology, Zhongshan Hospital, Fudan University, 200032 Shanghai, China; ^2^Shanghai Geriatric Medical Center, 201100 Shanghai, China; ^3^Shanghai Medical Center for Kidney Disease, Shanghai Municipal Health Commission, 200032 Shanghai, China; ^4^Shanghai Institute of Kidney and Dialysis, 200032 Shanghai, China; ^5^Hemodialysis Quality Control Center of Shanghai, Shanghai Medical Quality Control Management Center, 200032 Shanghai, China

**Keywords:** cardiovascular–kidney–metabolic syndrome, biomarkers, risk prediction, machine learning

## Abstract

Cardiovascular–kidney–metabolic (CKM) syndrome represents a complex interplay among cardiovascular disease, chronic kidney disease, and metabolic disorders; meanwhile, CKM syndrome affects a significant proportion of adults and increases the risk of heart failure, end-stage renal disease, and premature death. This review examines traditional and emerging biomarkers in CKM syndrome, focusing on the clinical utility and predictive value of these markers for disease progression and outcomes. Traditional markers include cardiovascular, renal, metabolic and inflammatory biomarkers. Meanwhile, emerging biomarkers include novel lipid biomarkers, epigenetic and microRNA markers, and metabolic and inflammatory markers. Composite tools integrating traditional and emerging biomarkers have shown promise for improving risk stratification and clinical decision-making. Moreover, the Predicting Risk of cardiovascular disease (CVD) Events (PREVENT) and Cardiovascular-Kidney-Metabolic 2-Stage 2-Biomarker Assessment Group (CKM_2_S_2_-BAG) equations have been developed for CKM syndrome risk stratification. Additionally, machine learning approaches are increasingly being applied in CKM syndrome research. Future research priorities include large-scale validation studies, standardization of biomarker measurement and interpretation, and the development of advanced technologies. The integration and validation of reliable biomarkers for CKM syndrome represent a critical step toward improving risk stratification and enabling personalized management strategies.

## 1. Introduction

Cardiovascular–kidney–metabolic (CKM) syndrome is a recently defined clinical entity that recognizes the interconnectedness of cardiovascular disease (CVD), chronic kidney disease (CKD), and metabolic disorders such as type 2 diabetes mellitus (T2DM) and obesity [1]. This syndrome represents a pathophysiologically integrated spectrum of conditions, rather than isolated organ-specific diseases, with shared mechanisms driving systemic inflammation, lipotoxicity, and fibrotic remodeling that collectively amplify adverse outcomes across organ systems [1]. This syndrome affects a substantial proportion of United States (US) adults and significantly increases the risk of heart failure, end-stage renal disease, and premature death [2].

The prevalence of CKM syndrome is substantial and growing globally, with population-level data indicating that nearly 90% of U.S. adults exhibit at least one stage of the CKM spectrum, and approximately one in three U.S. adults carry three or more key risk factors for the syndrome [2]. These risk factors—including obesity, T2DM, hypertension, and chronic kidney disease—act synergistically to accelerate disease progression, with risk escalating markedly across CKM stages 2 through 4 [1,2]. Emerging evidence highlights two critical modifiers of this risk gradient: first, significant racial and ethnic disparities, with Asian populations exhibiting distinct prevalence and mortality patterns compared to Western cohorts [3,4,5]; second, underrecognized hepatic involvement via metabolic dysfunction-associated steatotic liver disease (MASLD), which amplifies systemic inflammation and insulin resistance [1]. The American Heart Association’s recent scientific statements accordingly emphasize integrated, population-sensitive management approaches that address these multi-system interactions [1].

The identification and validation of reliable biomarkers for CKM syndrome are crucial for early detection, risk stratification, and personalized treatment strategies. Despite the robust scientific framework for CKM syndrome established by recent guidelines and reviews, critical gaps remain in its clinical implementation, particularly regarding early prevention, risk stratification, and tailored management approaches that account for the full spectrum of interconnected diseases [1,2]. Traditional biomarkers have been extensively used in clinical practice, but their limitations in capturing the complex interactions between organ systems have led to the development of novel biomarkers that may provide incremental prognostic value.

## 2. Traditional Biomarkers in CKM Syndrome

### 2.1 Cardiovascular Biomarkers

Traditional cardiovascular biomarkers in CKM syndrome include indicators of cardiac function, vascular health, and cardiovascular risk. Cardiac troponins (cardiac troponin T (cTnT), cardiac troponin I (cTnI)) are highly sensitive and specific markers of myocardial injury, with established roles in acute coronary syndrome diagnosis and risk stratification [6]. These markers have evolved from their original use in acute myocardial infarction to applications in chronic disease states, including CKM syndrome, where they may reflect ongoing myocardial stress and injury. Brain natriuretic peptide (BNP) and N-terminal pro-BNP (NT-proBNP) are essential biomarkers for heart failure diagnosis and prognosis, with well-established clinical utility [7]. The European Society of Cardiology (ESC) guidelines provide clear recommendations for using these biomarkers in heart failure management—a consideration particularly relevant to CKM syndrome, given the high prevalence of concurrent cardiac dysfunction in this population [8]. However, despite extensive validation for isolated cardiovascular risk assessment, the utility of these biomarkers in CKM syndrome may be constrained by the complex interactions between cardiovascular, kidney, and metabolic systems.

### 2.2 Kidney Function Biomarkers

Kidney function biomarkers include estimated glomerular filtration rate (eGFR) and albuminuria [9]. These biomarkers are essential for identifying and monitoring kidney dysfunction in CKM syndrome. eGFR is calculated by serum creatinine or cystatin C, or by renogram. Serum creatinine remains the cornerstone of kidney function assessment, while cystatin C provides an alternative marker that is less influenced by muscle mass and diet [9]. The development of new creatinine- and cystatin C-based equations to estimate GFR without race has improved the accuracy of kidney function assessment and addressed important concerns about racial bias in clinical algorithms [9]. Renogram can detect the function of each kidney. Albuminuria and urine albumin-creatinine ratio (UACR) serve as a sensitive marker of kidney damage and cardiovascular risk [10]. Claudel and Verma [10] provided a comprehensive state-of-the-art review of albuminuria in cardiovascular, kidney, and metabolic disorders, highlighting its role as a key biomarker that reflects both kidney damage and systemic vascular dysfunction. These biomarkers may not adequately reflect the early stages of kidney injury or the complex interactions between kidney function and other organ systems. There are also studies of kidney tubular biomarkers over the past decades, such as urinary kidney injury molecule-1 (KIM-1) and neutrophil gelatinase-associated lipocalin (NGAL), which can indicate the early kidney injury [11,12]. However, these novel biomarkers have not been applied in clinical practice.

### 2.3 Metabolic Biomarkers

Metabolic biomarkers include body mass index (BMI), waist circumference (WC), Waist-to-Height Ratio (WHtR), glucose, Hemoglobin A1c (HbA1c), and lipid profiles [13,14,15]. These biomarkers are critical for identifying metabolic dysfunction in CKM syndrome. BMI ≥25 kg/m^2^ (or ≥23 kg/m^2^ if Asian ancestry) and WC ≥88/102 cm in women/men (or if Asian ancestry ≥80/90 cm in women/men) define CKM syndrome Stage 1, indicating excess or dysfunctional adiposity [1]. Approximately 81% of subjects present with both elevated BMI and WC; however, 21.7% of individuals with normal BMI but increased WC—representing abdominal obesity—demonstrate worse metabolic profiles [16,17]. WHtR is shown to be associated with CKM syndrome in the Metabolic Syndrome in the Community of Madrid Study (SIMETAP) study [18]. These findings highlight central obesity as a critical marker for disease progression in CKM. Glucose and HbA1c provide essential information about glycemic control, while insulin resistance indices help assess metabolic dysfunction [13]. Lipid profiles, including traditional measures such as total cholesterol, low-density lipoprotein cholesterol (LDL-C), high-density lipoprotein cholesterol (HDL-C), and triglycerides, remain fundamental for cardiovascular risk assessment [14,15]. What’s more, studies have shown that obesity and subsequent insulin resistance lead to dyslipidemia and glucose homeostasis disorder, indicating the interaction effect between these metabolic biomarkers in CKM syndrome [14,15]. However, these biomarkers may not capture the full spectrum of metabolic derangements or their interactions with cardiovascular and kidney systems.

### 2.4 Inflammatory Factors

Systemic inflammation is crucial in driving the progression of CKM syndrome stages. Inflammatory factors such as C-reactive protein (CRP) and interleukin-6 (IL-6), and tumor necrosis factor (TNF)-α are shown to be associated with CKM syndrome [19,20]. CRP and high-sensitivity CRP (hs-CRP) have been sufficiently studied to predict cardiovascular risk and outcomes [21,22]. In the Multi-Ethnic Study of Atherosclerosis (MESA), participants with hs-CRP over 2 mg/L had shorter atherosclerotic cardiovascular disease (ASCVD)- and heart failure (HF)-free survival [22]. CRP is indicated to be associated with abnormal renal function, dyslipidemia, diabetes and metabolic syndrome [23,24], and is also shown to cause renal fibrosis via Smad3-dependent pathways [25]. IL-6 and TNF-α have been shown to be associated with obesity, T2DM, CKD, CVD progression in Chronic Renal Insufficiency Cohort (CRIC) Study [26,27,28,29]. A recent study has also shown IL-6 was associated with heart failure and CVD in CKM syndrome [22].

### 2.5 Hepatic Biomarkers

The liver constitutes a central metabolic hub in CKM syndrome, with MASLD representing both a consequence and amplifier of systemic metabolic derangements [30,31]. Serum alanine aminotransferase (ALT) and aspartate aminotransferase (AST), along with gamma-glutamyl transferase (GGT), remain standard screening tools, though they lack specificity for hepatic steatosis [32]. Non-invasive composite scores—namely the Fibrosis-4 (FIB-4) index and NAFLD Fibrosis Score (NFS)—integrate routine clinical parameters to estimate fibrosis risk, have been associated with the risk of developing diabetes mellitus, cardiovascular disease, and chronic kidney disease, highlighting their role in broader metabolic risk stratification [33]. These hepatic biomarkers face standardization challenges analogous to other CKM composite tools, including ethnic variation in fibrosis thresholds and dynamic fluctuations during weight change [1].

## 3. Emerging Biomarkers in CKM Syndrome

### 3.1 Novel Lipid Biomarkers

Novel lipid biomarkers, such as Lipoprotein(a) [Lp(a)], leptin and adiponectin, and lipid metabolomics, have demonstrated strong associations with cardiovascular disease risk and CKM syndrome progression [34,35,36,37]. Lp(a) is a LDL-like particle and its blood levels are mainly determined by genetic factors [38]. Lp(a) has been shown to be associated with CVD events and can predict CVD in studies of recent years [39,40,41]. Statins and ezetimibe are ineffective and proprotein convertase subtilisin/kexin type 9 (PCSK9) inhibitors have modest reduction to Lp(a). Therapies targeting Lp(a) are now being studied in several trials with cardiovascular outcome such as HORIZON, OCEAN and ACCLAIM [38,42]. Leptin and adiponectin are factors secreted by adipose tissues. Leptin acts as a pro-inflammatory while adiponectin as an anti-inflammatory adipokine [43]. Studies have shown the application of leptin and adiponectin in obesity, T2DM, CKD and CVD [34,44,45]. The leptin-adiponectin ratio (Adpn/Lep) is shown to be a biomarker in insulin resistance and CVD [46,47]. Recently, lipid metabolomics are used to identify CKM syndrome [35,48,49]. Li et al. [50] identified mitochondrial lactamase beta (LACTB)-mediated lipid metabolism as a key mechanism in CKM pathogenesis. One study found that substantial metabolic heterogeneity was observed in patients with CKM syndrome stage 3 and a panel of 20 metabolites showed high diagnostic efficacy for CKM syndrome stage 3 (area under the curve (AUC) = 0.875) [35]. However, lipid metabolomics remains limited by high costs and lack of validation. Lipid combined biomarkers containing triglyceride and HDL-C, can serve as a novel tool for insulin resistance (Section 4.1 for details). These novel lipid biomarkers provide additional value for risk stratification in CKM syndrome management.

### 3.2 Epigenetic and MicroRNAs Biomarkers

Epigenetic modifications, particularly DNA methylation age, demonstrate mortality predictive capacity in CKM patients beyond conventional risk factors [51]. Wu et al. [51] specifically investigated DNA methylation age acceleration among US adults with CKM syndrome, confirming that epigenetic aging provides prognostic information incremental to traditional risk factors. These epigenetic indicators may offer valuable perspectives on biological aging processes and disease progression in CKM syndrome, representing a novel approach to understanding the biological mechanisms underlying CKM syndrome progression and outcomes.

Beyond epigenetic modifications, microRNAs (miRNAs) act as critical post-transcriptional regulators in CKM syndrome; their dysregulation contributes to inflammation, fibrosis, and cardiovascular dysfunction [52,53,54]. Recent studies have implicated several miRNAs in cardiovascular and metabolic processes relevant to CKM syndrome, including microRNA-199a-3p (miR-199a-3p) [55], microRNA-199a-5p (miR-199a-5p) [56], microRNA-126 (*miR-126*) [57,58], microRNA-423-5p (*miR-423-5p*) [59,60], and circulating small extracellular vesicle miRNAs [61,62]. However, these biomarkers require validation in large-scale prospective cohort studies before clinical implementation.

### 3.3 Novel Metabolic and Inflammation Biomarkers

Recent studies have identified novel metabolic and inflammatory biomarkers that may provide additional value in CKM syndrome assessment. Kipp et al. [63] demonstrated that bilirubin bioconversion in the gut-liver-kidney axis serves as a novel biomarker for insulin resistance in CKM syndrome. Soluble urokinase plasminogen activator receptor (suPAR), an inflammatory marker, has been shown to be related to systemic chronic inflammation, T2DM, CKD, and CVD [64,65,66]. Galectin-3 (Gal-3) is also showing promise for chronic inflammation, fibrosis, and progression of renal/vascular disease in CKM patients [67,68,69]. Growth Differentiation Factor-15 (GDF-15) has emerged as a particularly promising biomarker [70]. GDF-15 may provide additional prognostic value beyond traditional biomarkers, particularly in identifying patients at high risk for adverse outcomes. Advanced technologies are being explored for CKM syndrome research and management. Brown and Huck discussed the potential utility of total-body PET imaging for investigating mechanisms in CKM syndrome [71].

## 4. Applications and Risk Stratification

### 4.1 Metabolic and Inflammatory Composite Tools

Metabolic and inflammatory composite biomarkers have emerged as promising tools for CKM syndrome risk assessment, providing superior predictive value compared to individual metabolic parameters. Insulin resistance indices have been widely used in CKM syndrome [72,73]. Insulin resistance indices including several methods such as homeostatic model assessment for insulin resistance (HOMA-IR), quantitative insulin sensitivity check index (QUICKI), metabolic score for insulin resistance (METS-IR), or triglyceride-glucose (TyG) [74,75,76,77]. Estimated glucose disposal rate (eGDR) has also been used as insulin resistance indices in CKM syndrome [78,79]. The atherogenic index of plasma (AIP) has been shown to correlate with cardiovascular disease risk across different CKM syndrome stages in nationwide prospective cohort studies [36,37]. Elevated AIP levels have been associated with increased future risk of cardiovascular disease in individuals with CKM syndrome stages 0–3 [36]. The stress hyperglycemia ratio (SHR), reflecting acute glycemic stress, has demonstrated significant prognostic value for mortality risk across CKM stages [80,81]. In a recent study using National Health and Nutrition Examination Survey (NHANES) data, elevated SHR was associated with increased mortality risk in CKM syndrome stages 0–3, with the highest quartile showing Hazard Ratio (HR) = 1.19 (95% CI = 1.03–1.37) for all-cause mortality compared to the lowest quartile, and there is a U-shaped relationship between SHR and mortality, with optimal SHR values of 0.89 for all-cause mortality and 0.91 for cardiovascular mortality [81].

There are TyG-related biomarkers for insulin resistance and have been applied in the CKM syndrome [82]. The triglyceride-glucose-body mass index (TyG-BMI) demonstrates strong predictive value for cardiovascular disease risk and mortality, with elevated levels significantly associated with increased CVD risk across CKM syndrome stages 0–3 (hazard ratios 1.15–1.89) and showing a 2.3-fold increased mortality risk in the highest versus lowest quartile in the China Health and Retirement Longitudinal Study (CHARLS) cohort [83,84]. Other TyG-related Indices such as triglyceride-glucose-waist-to-height ratio (TyG-WHtR), triglyceride-glucose-waist circumference (TyG-WC), triglyceride-glucose-body roundness index (TyG-BRI), triglyceride-glucose-a body shape index (TyG-ABSI), triglyceride-glucose-weight-adjusted waist index (TyG-WWI), triglyceride-glucose-Chinese visceral adiposity index (TyG-CVAI), triglyceride-glucose-neck circumference (TyG-NC), triglyceride-glucose-neck-to-height ratio (TyG-NHtR), and cumulative triglyceride-glucose (cumTyG) are also shown to be correlated with all-cause mortality in CKM syndrome by cohort such as NHANES and CHARLS database [83,85,86,87,88].

Composite indicators for inflammation have also been reported. The C-reactive protein-triglyceride glucose index (CTI) level is correlated with the all-cause death risk in patients with CKM stages 0–3 in the CHARLS database [89]. Systemic immune-inflammation index (SII, SII = Platelet count × Neutrophil count/Lymphocyte count), a composite marker integrating neutrophil, lymphocyte, and platelet counts, has shown significant association with CKM syndrome and may provide additional value in risk stratification [90]. Oxidative balance score (OBS) is a composite measure that integrates dietary and lifestyle factors associated with oxidative stress [91]. Chen et al. [92] demonstrated that OBS significantly predicts CKM progression and mortality risk, with higher OBS associated with reduced risk of adverse outcomes. Xie et al. [93] confirmed these findings in a large population-based study, showing that OBS independently predicts CKM syndrome progression and mortality. The OBS provides a comprehensive assessment of oxidative stress status and may be particularly useful for risk stratification in CKM syndrome. These composite biomarkers provide enhanced risk stratification capabilities and may guide personalized treatment strategies in CKM syndrome management. Detailed formulas for these metabolic and inflammatory composite tools are summarized in Table 1 (Ref. [1,18,36,74,76,77,79,85,86,87,89,90,94,95,96,97,98,99]).

**Table 1.** **Metabolic and inflammatory composite tools**.

Index	Formula	References
HOMA-IR	$\frac{Fasting\;Insulin\;[\mu U/mL]\times FBG\;[mg/dL]}{405}$	[74]
QUICKI	$\frac{1}{lg(Fasting\;Insulin\;[\mu U/mL])+lg(FBG\;[mg/dL])}$	[74]
METS-IR	$\frac{ln\left(2\times FBG\;[mg/dL]+TG\;[mg/dL]\right)\times BMI}{ln\left(HDL\text{-}C\;[mg/dL]\right)}$	[77]
eGDR (based on WC)	21.158−(0.09×WC [cm])−(3.407×HTN)−(0.551×HbA1c [%])	[79]
BMI	$\frac{Weight\;[kg]}{Height\;[m{]}^{2}}$	[1]
WHtR	$\frac{WC\;[cm]}{Height\;[cm]}$	[18]
BRI	$364.2-365.5\times \sqrt{1-{\left(\frac{WC\;\left[cm\right]/2\pi }{\left(0.5\times Height\;\left[cm\right]\right)}\right)}^{2}}$	[95]
ABSI	$\frac{WC\;[cm]}{BMI\;[kg/m^{2}{]}^{2/3}\times Height\;[cm{]}^{1/2}}$	[96]
WWI	$\frac{WC\;[cm]}{\sqrt{Weight\;[kg]}}$	[97]
TyG-BRI	*TyG/BRI*	[85]
TyG-ABSI	*TyG/ABSI*	[85]
TyG-WWI	*TyG/WWI*	[85]
CVAI (for males)	$-267.93+0.68\times Age\;[yr]+0.03\times BMI\;[kg/m^{2}]+4.00\times WC\;[cm]+22.00\times lg(TG\;[mmol/L])-16.32\times HDL\text{-}C\;[mmol/L]$	[98]
CVAI (for females)	−187.32+1.71×Age [yr]+4.23×BMI+1.12×WC [cm]+39.76×lg(TG [mmol/L])−11.66×HDL-C [mmol/L]	[98]
AIP	$Lg\left(\frac{TG\;[mg/dL]}{HDL\text{-}C\;[mg/dL]}\right)$	[36]
SHR	$\frac{FBG\;[mg/dL]}{28.7\times HbA1c\;\left(\%\right)-46.7}$	[99]
TyG	Ln(TG [mg/dL]×FBG [mg/dL]/2)	[76]
cumTyG	*Mean TyG × time span [years], such as (TyG2012 + TyG 2015)/2 × (2015–2012)*	[86]
TyG-BMI	*TyG/BMI*	[94]
TyG-WC	*TyG/WC*	[94]
TyG-WHtR	*TyG/WHtR*	[94]
TyG-CVAI	*TyG/CVAI*	[85]
TyG-NC	*TyG/NC*	[87]
TyG-NHtR	$\frac{TyG}{NC\;[cm]/height\;[cm]}$	[87]
CTI	0.412×ln(CRP [mg/L])+TyG	[89]
SII	$\frac{Platelet\;count\;[10^{9}/L]\times Neutrophil\;count\;[10^{9}/L]}{Lymphocyte\;count\;[10^{9}/L]}$	[90]

Note: TyG was calculated as ln[TG (mg/dL) × FBG (mg/dL)/2], and cumTyG was calculated as the mean TyG multiplied by the time span (years). BRI, ABSI, WWI, CVAI, and the TyG-derived indices were calculated according to the formulas and original definitions shown in Table 1. FBG, fasting blood glucose; TC, total cholesterol; HDL-C, high-density lipoprotein cholesterol; NHtR, neck circumference to height ratio; WC, waist circumference; HTN, hypertension status (0 = normotensive, 1 = hypertensive by medication or BP ≥140/90 mmHg); eGDR, estimated glucose disposal rate; HbA1c, hemoglobin A1c; BRI, body roundness index; cumTyG, cumulative of TyG; ABSI, a body shape index; WWI, weight-adjusted waist index; CVAI, Chinese visceral adiposity index; NC, neck circumference; WHtR, Waist-to-Height Ratio; AIP, atherogenic index of plasma; SHR, stress hyperglycemia ratio; CTI, C-reactive protein-triglyceride glucose index; CRP, C-reactive protein; SII, systemic immune-inflammation index; HOMA-IR, homeostatic model assessment for insulin resistance; QUICKI, quantitative insulin sensitivity check index; METS-IR, metabolic score for insulin resistance; BMI, body mass index; TyG, triglyceride-glucose; TyG-NC, triglyceride-glucose-neck circumference; TG, triglyceride.

Despite the proliferating number of metabolic and inflammatory composite tools, their clinical translation remains constrained by several standardization barriers. Firstly, cut-off values for indicators like the TyG index vary across populations, with ethnic-specific thresholds potentially needed [3,4,100]. Secondly, biomarker measurement itself lacks standardization; fasting glucose and triglyceride levels are influenced by pre-analytical factors like fasting duration and diet. Thirdly, inconsistencies in coding categorical variables (e.g., hypertension definitions in eGDR formulas) introduce misclassification risks. These gaps underscore the need for international consensus on harmonized reference ranges. Until then, composite tools should supplement—not replace—clinical judgment, with results interpreted within population-specific contexts.

### 4.2 Risk Prediction

Multiple biomarker integration approaches have been developed to enhance outcome prediction in CKM syndrome. These composite scoring systems may offer superior risk assessment capabilities when compared to conventional risk factor evaluation. The Predicting Risk of CVD Events (PREVENT) and CKM_2_S_2_-BAG are specifically developed for CKM syndrome. The PREVENT equations developed by the American Heart Association are sex-specific and race-free and provide 10- and 30-year risk estimates for CVD in CKM syndrome [101]. The base equations include age, sex, blood pressure, cholesterol, medication use, diabetes, tobacco use, and kidney function, while the additional equations include social determinants of health (SDOH), UACR, and HbA1c. The CKM_2_S_2_-BAG score, incorporating cholesterol, kidney function, sex, smoking, blood pressure, age, and glucose, has demonstrated good-to-excellent performance in cardiovascular risk stratification and discrimination between low-, intermediate-, and high-risk individuals [102]. However, despite their promise, the clinical translation of these tools faces several implementation barriers. These include: (1) lack of standardized biomarker assays and population-agnostic reference ranges; (2) uncertain cost-effectiveness and reimbursement pathways; (3) variable predictive performance across ethnic and age subgroups (e.g., differential accuracy in Asian versus European cohorts); and (4) limited applicability in resource-constrained settings without advanced laboratory infrastructure. Addressing these gaps through assay harmonization, health economic evaluations, and multi-ethnic validation studies is essential to ensure equitable and widespread clinical adoption.

### 4.3 The Pathophysiological Pathways of Biomarkers

Fig. 1 provides an integrative framework summarizing the biomarkers discussed in this review. The pathophysiological pathways of CKM syndrome commonly originates from excess or dysfunctional adipose tissue. Metabolic biomarkers such as BMI, WC, WHtR and lipid biomarkers including total cholesterol (TC), LDL-C, Lp(a), oxidized low-density lipoprotein (xLDL), leptin, and adiponectin reflect the abnormal adipose tissue. Dysfunctional adipose tissue (especially visceral adipose tissue) secretes proinflammatory cytokines and prooxidative products mediators including CRP, IL-6, TNF-α, suPAR, Gal-3, GDF-15. And the inflammatory processes can be estimated by inflammatory composite tools such as CTI, OBS, and SII. The epigenetic and microRNAs biomarkers such as *miR-199a-3p*, *miR-126*, and *miR-423-5p *may early response for this stage. Inflammatory processes result in insulin resistance, which can be reflected by glucose, HbA1c, SHR, AIP, insulin resistance indices, and TyG-related indices. The development of MASLD amplifies metabolic inflammation. The pro-oxidative and proinflammatory mediators cause atherosclerosis, CVD, and CKD. The biomarkers of CVD and CKD reflect the injury (section 2.1 and 2.2).

**Fig. 1.**
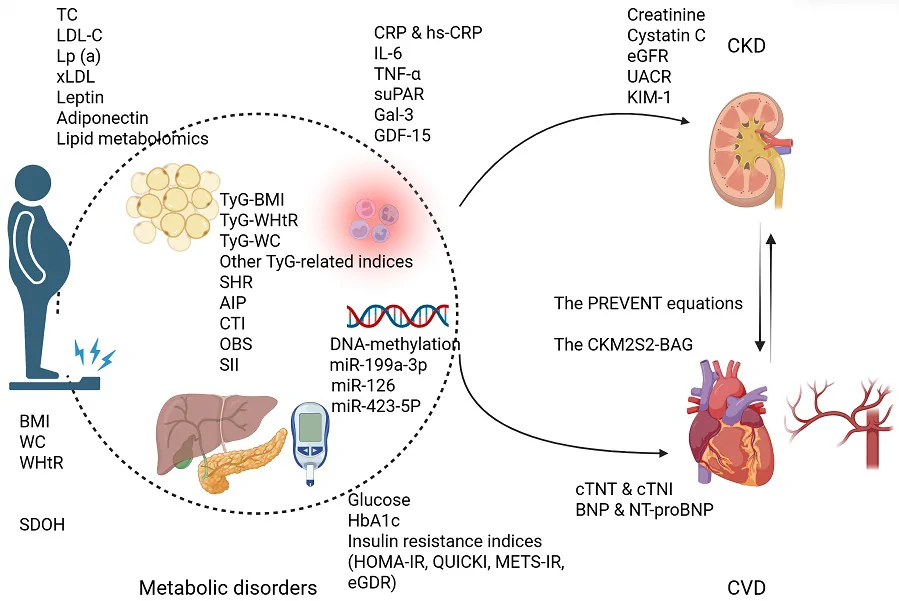
**Biomarkers and **a**pplications in CKM syndrome**. AIP, atherogenic index of plasma; BMI, body mass index; BNP, B-type natriuretic peptide; CKD, chronic kidney disease; CKM, cardiovascular-kidney-metabolic; CRP, C-reactive protein; CTI, C-reactive protein-triglyceride glucose index; CVD, cardiovascular disease; eGDR, estimated glucose disposal rate; eGFR, estimated glomerular filtration rate; Gal-3, Galectin-3; GDF-15, growth differentiation factor-15; HbA1c, hemoglobin A1c; hs-CRP, high-sensitivity C-reactive protein; IL-6, interleukin-6; Lp(a), lipoprotein(a); NT-proBNP, N-terminal pro-B-type natriuretic peptide; OBS, oxidative balance score; SDOH, social determinants of health; SHR, stress hyperglycemia ratio; SII, systemic immune-inflammation index; suPAR, soluble urokinase plasminogen activator receptor; TNF, tumor necrosis factor; TyG, triglyceride-glucose; UACR, urine albumin-creatinine ratio; WC, waist circumference; WHtR, waist-to-height ratio; LDL-C, low-density lipoprotein cholesterol; xLDL, oxidized low-density lipoprotein; miR-126, microRNA-126; miR-199a-3p, microRNA-199a-3p; miR-423-5p, microRNA-423-5p; PREVENT, Predicting Risk of CVD Events; CKM_2_S_2_-BAG, Cardiovascular-Kidney-Metabolic 2-Stage 2-Biomarker Assessment Group; KIM-1, kidney injury molecule-1.

## 5. Future Directions and Challenges

### 5.1 Validation, Standardization, and Implementation

Robust validation frameworks are fundamental for translating emerging biomarkers from research to clinical practice. Large-scale validation studies are required to confirm the clinical utility and prognostic accuracy of novel biomarkers in CKM syndrome across diverse patient populations. Prospective studies with extended follow-up periods are critical to establish the long-term prognostic value and temporal stability of these markers [36,51]. Additionally, multicenter validation initiatives are vital to ensure generalizability across different healthcare systems and demographic populations. Following validation, the successful integration of biomarker-based approaches into routine clinical practice requires comprehensive standardization of measurement protocols, interpretation criteria, and implementation strategies. Standardization of biomarker measurement and interpretation is pivotal for widespread clinical implementation, ensuring consistency and reliability across different healthcare settings [8]. The development of evidence-based guidelines for biomarker use in CKM syndrome management is indispensable to provide clinicians with clear recommendations for patient assessment, risk stratification, and treatment decision-making. Such guidelines should encompass establishing reference ranges, cutoff values, and clinical decision thresholds applicable across diverse patient populations.

### 5.2 The Role of the Liver

Increasing evidence suggests that MASLD or metabolic dysfunction-associated fatty liver disease (MAFLD), formerly known as NAFLD, constitutes a component of CKM syndrome [30,31,103,104,105,106]. Some researchers have expanded the CKM syndrome concept to include hepatic involvement, proposing the Cardiovascular-Renal-Hepatic-Metabolic (CRHM) syndrome [107]. The diagnosis of MASLD requires the presence of hepatic steatosis (typically detected by ultrasonography) combined with ≥1 typical features of the metabolic syndrome (abdominal overweight/obesity, prediabetes/type 2 diabetes, hypertension, hypertriglyceridemia, or low HDL cholesterol), along with alcohol consumption below sex-specific thresholds (<140 g/week for women; <210 g/week for men), after exclusion of other known causes of hepatic steatosis. In addition to histological examinations, non-invasive biomarkers are under development. Novel therapeutics targeting MASLD may consequently confer benefits for CKM syndrome. Resmetirom, a liver-directed, thyroid hormone receptor β-selective agonist, and semaglutide, a glucagon-like peptide-1 receptor agonist, have been conditionally approved by the US Food and Drug Administration for the MASLD. Moreover, a phase 2b clinical trial of efruxifermin—a novel fibroblast growth factor 21 analog—demonstrated not only reduction in MASLD-related fibrosis but also significant improvements in glycemic and lipid parameters [108].

### 5.3 Social Determinants and Health Behaviors

Recent research has highlighted the importance of SDOH and health behaviors in CKM syndrome. Evidence suggests that health behavior scores, such as Life’s Essential 8, are closely associated with prognosis in CKM syndrome patients [109]. Additionally, social determinants including socioeconomic status and environmental factors significantly influence the prevalence and outcomes of CKM syndrome [110]. These findings emphasize that beyond traditional biomedical factors, addressing social determinants and promoting healthy behaviors are fundamental for comprehensive risk reduction and improved outcomes in CKM syndrome prevention and management.

### 5.4 Machine Learning

Machine learning approaches are being increasingly applied to CKM syndrome research, with multiple studies demonstrating their value in risk prediction and biomarker analysis. Machine learning approaches can be categorized into supervised learning and unsupervised learning. Supervised learning uses selected, processed, and weighted features to identify the optimal combination for specific outcomes, while unsupervised learning explores patterns in data without predefined labels [111].

The superiority of machine learning in CKM syndrome stems from its ability to handle the syndrome’s inherent complexity—high-dimensional, non-linear interactions among metabolic, inflammatory, and hemodynamic biomarkers that traditional regression models cannot capture. This improvement underscores the capacity of machine learning algorithms to capture complex, non-linear relationships among variables that traditional models may overlook. Moreover, machine learning models can effectively integrate clinical data, electronic health records (EHRs), imaging data, and novel biomarkers, thereby enhancing predictive accuracy. A systematic review and meta-analysis comparing machine-learning versus traditional approaches for atherosclerotic cardiovascular risk prognostication in 16 studies found that machine-learning models have higher summary C-statistics than traditional risk scores (0.773 vs. 0.759) [112]. One study found that electronic health records yielded better predictions for coronary artery disease than the pooled cohort equations using machine learning including random forest, gradient boosted trees, and support vector machine in BioMe Biobank and UK Biobank [113]. Studies also combine coronary computed tomography or echocardiography with other parameters to show that machine learning can predict the CVD events or death and the models are better than traditional prediction models [114,115]. Several recent studies have used machine learning to investigate the predictive role of novel biomarkers in CKM syndrome [92,116,117,118,119,120]. Given that the CKM represents a multi-factor clinical syndrome that encompasses multiple organs and systems, exhibits high heterogeneity, and is associated with numerous biomarkers, machine learning techniques can play a pivotal role in risk prediction and treatment prognosis evaluation. These computational approaches enable the integration of multiple biomarkers and clinical parameters to develop more accurate prediction models for CKM syndrome progression and outcomes.

## 6. Conclusion

The identification and validation of reliable biomarkers for CKM syndrome represent a critical step toward improved risk stratification and personalized management strategies. While traditional biomarkers remain important for routine clinical assessment, emerging biomarkers show promise in providing additional prognostic and predictive value. The integration of multiple biomarkers may enable more accurate risk assessment and guide personalized treatment strategies, ultimately improving patient outcomes and reduced disease burden. Future research should focus on large-scale validation studies across diverse populations, standardization of biomarker implementation in clinical practice, and the incorporation of advanced technologies such as machine learning and artificial intelligence to further enhance predictive accuracy and clinical utility.

## Abbreviations

ABSI, a body shape index; AIP, atherogenic index of plasma; ASCVD, atherosclerotic cardiovascular disease; BMI, body mass index; BNP, B-type natriuretic peptide; BRI, body roundness index; CHARLS, China Health and Retirement Longitudinal Study; CKD, chronic kidney disease; CKM, cardiovascular-kidney-metabolic; CKM_2_S_2_-BAG, Cardiovascular-Kidney-Metabolic 2-Stage 2-Biomarker Assessment Group; CRHM, cardiovascular-renal-hepatic-metabolic; CRIC, Chronic Renal Insufficiency Cohort; CRP, C-reactive protein; CTI, C-reactive protein-triglyceride glucose index; CVD, cardiovascular disease; CVAI, Chinese visceral adiposity index; cTnI, cardiac troponin I; cTnT, cardiac troponin T; cumTyG, cumulative triglyceride-glucose; eGDR, estimated glucose disposal rate; eGFR, estimated glomerular filtration rate; ESC, European Society of Cardiology; FBG, fasting blood glucose; Gal-3, galectin-3; GDF-15, growth differentiation factor-15; HbA1c, hemoglobin A1c; HDL-C, high-density lipoprotein cholesterol; HF, heart failure; HOMA-IR, homeostatic model assessment for insulin resistance; hs-CRP, high-sensitivity C-reactive protein; IL-6, interleukin-6; KIM-1, kidney injury molecule-1; LDL-C, low-density lipoprotein cholesterol; Lp(a), lipoprotein(a); MAFLD, metabolic dysfunction-associated fatty liver disease; MESA, Multi-Ethnic Study of Atherosclerosis; METS-IR, metabolic score for insulin resistance; miR-126, microRNA-126; miR-199a-3p, microRNA-199a-3p; miR-423-5p, microRNA-423-5p; miRNAs, microRNAs; NC, neck circumference; NGAL, neutrophil gelatinase-associated lipocalin; NHANES, National Health and Nutrition Examination Survey; NHtR, neck-to-height ratio; NT-proBNP, N-terminal pro-B-type natriuretic peptide; OBS, oxidative balance score; PCSK9, proprotein convertase subtilisin/kexin type 9; PREVENT, Predicting Risk of CVD Events; QUICKI, quantitative insulin sensitivity check index; SDOH, social determinants of health; SHR, stress hyperglycemia ratio; SII, systemic immune-inflammation index; SIMETAP, Síndrome Metabólica en población de la Comunidad de Madrid (Metabolic Syndrome in the Community of Madrid Study); suPAR, soluble urokinase plasminogen activator receptor; T2DM, type 2 diabetes mellitus; TC, total cholesterol; TG, triglyceride; TNF-α, tumor necrosis factor-alpha; TyG, triglyceride-glucose; TyG-ABSI, triglyceride-glucose-a body shape index; TyG-BMI, triglyceride-glucose-body mass index; TyG-BRI, triglyceride-glucose-body roundness index; TyG-CVAI, triglyceride-glucose-Chinese visceral adiposity index; TyG-NC, triglyceride-glucose-neck circumference; TyG-NHtR, triglyceride-glucose-neck-to-height ratio; TyG-WC, triglyceride-glucose-waist circumference; TyG-WHtR, triglyceride-glucose-waist-to-height ratio; TyG-WWI, triglyceride-glucose-weight-adjusted waist index; UACR, urine albumin-creatinine ratio; US, United States; WC, waist circumference; WHtR, waist-to-height ratio; WWI, weight-adjusted waist index; xLDL, oxidized low-density lipoprotein.
